# Partial-Order Reduction for Parity Games with an Application on Parameterised Boolean Equation Systems

**DOI:** 10.1007/978-3-030-45237-7_19

**Published:** 2020-03-13

**Authors:** Thomas Neele, Tim A. C. Willemse, Wieger Wesselink

**Affiliations:** 8 Johannes Kepler University, Linz, Austria; 9 University of Birmingham, Birmingham, UK; Eindhoven University of Technology, Eindhoven, The Netherlands

## Abstract

Partial-order reduction (POR) is a well-established technique to combat the problem of state-space explosion. We propose POR techniques that are sound for parity games, a well-established formalism for solving a variety of decision problems. As a consequence, we obtain the first POR method that is sound for model checking for the full modal $\mu$-calculus. Our technique is applied to, and implemented for the fixed point logic called *parameterised Boolean equation systems*, which provides a high-level representation of parity games. Experiments indicate that substantial reductions can be achieved.

## 1 Introduction

In the field of formal methods, model checking [2] is a popular technique to analyse the behaviour of concurrent processes. However, the arbitrary interleaving of these parallel processes can cause an exponential blowup, which is known as the *state-space explosion* problem. Several approaches have been identified to alleviate this issue, by reducing the state space *on-the-fly*, *i.e.*, while generating it. Two established techniques are *symmetry reduction* [13] and *partial-order reduction* (POR) [8,26,30]. Whereas symmetry reduction can only be applied to systems that contain several copies of a component, POR also applies to heterogeneous systems. However, a major drawback of POR is that most variants at best preserve only a fragment of a given logic, such as LTL or CTL* without the next operator ($\text{LTL}_{-X}/\text{CTL}^*_{-X}$) [7] or the weak modal $\mu$-calculus [28]. Furthermore, the variants of POR that preserve a branching time logic impose significant restrictions on the reduction by only allowing the prioritisation of exactly one action at a time. This decreases the amount of reduction achieved.

In this paper, we address these shortcomings by applying POR on parity games. A parity game is an infinite-duration, two-player game played on a directed graph with decorations on the nodes, in which the players *even* (denoted $\Diamond$) and *odd* (denoted $\Box$) strive to win the nodes of the graph. An application of parity games is encoding a model checking question: a combination of a model, in the form of a transition system, and a formal property, formulated in the modal $\mu$-calculus [16]. In such games, every node $v$ represents the combination of a state $s$ from the transition system and a (sub)formula $\varphi$. Under a typical encoding, player $\Diamond$ wins in $v$ if and only if $\varphi$ holds in $s$.

In the context of model checking, parity games suffer from the same state-space explosion that models do. Exploring the state space of a parity game under POR can be a very effective way to address this. Our contributions are as follows:

- We propose conditions (Def. 4) that ensure that the *reduction function* used to reduce the parity game is correct, *i.e.*, preserves the winning player of the parity game (Thm. 1).
- We identify improvements for the reduction by investigating the typical structure of a parity game that encodes a model checking question.
- We illustrate how to apply our POR technique in the context of solving *parameterised Boolean equation systems* (PBESs) [10]—a fixed point logic closely related to LFP—as a high-level representation of a parity game.
- We extend the ideas of [17] with support for non-determinism and experiment with an implementation for solving PBESs.

Our approach has two distinct benefits over traditional POR techniques that operate on transition systems. First, it is the first work that enables the use of partial-order reduction for model checking for the full modal $\mu$-calculus. Second, the conditions that we propose are strictly weaker than those necessary to preserve the branching structure of a transition system used in other approaches to POR for branching time logics [7,28], increasing the effectiveness of POR.

The experiments with our implementation for solving PBESs are quite promising. Our results show that, in particular, those instances in which PBESs encode model checking problems involving large state spaces benefit from the use of partial-order reduction. In such cases, a significant size reduction is possible, even when checking complex $\mu$-calculus formulae, and the time penalty of conducting the static analysis is more than made up for by the speed-up in the state space exploration phase.

*Related Work* There are several proposals for using partial-order reduction for branching-time logics. Groote and Sellink [9] define several forms of *confluence reduction* and prove which behavioural equivalences (and by extension, which fragments of logics) are preserved. In confluence reduction, one tries to identify internal transitions that can safely be prioritised, leading to a smaller state space. Ramakrishna and Smolka [28] propose a notion that coincides with strong confluence from [9], preserving weak bisimilarity and the corresponding logic weak modal $\mu$-calculus.

Similar ideas are presented by Gerth *et al.* in [7]. Their approach is based on the *ample set* method [26] and preserves a relation they call visible bisimulation and the associated logic $\text{CTL}_{-X}$. To preserve the branching structure, they introduce a *singleton proviso* which, contrary to our theory, can greatly impair the amount of reduction that can be achieved (see our Example 3, page 313).

Valmari [33] describes the *stubborn sets* method for $\text{LTL}_{-X}$ model checking. In general, stubborn sets allow for larger reductions than ample sets. While investigating the use of stubborn sets for parity games, we identified a subtle issue in one of the stubborn set conditions (called **D1** in [33]). When applied to LTSs or KSs, this means that $\mathrm{LTL}_{-X}$ is not necessarily preserved. Moreover, using the condition in the setting of parity games may result in games with different winners; for an example, see our technical report [24]. In [21], we further explore the consequences of the faulty condition for stubborn-set based POR techniques that can be found in the literature. We here resort to a strengthened version of condition **D1** that does not suffer from these issues.

Similar to our approach, Peled [27] applies POR on the product of a transition system and a Büchi automaton, which represents an $\mathrm{LTL}_{-X}$ property. It is important to note, though, that this original theory is not sound, as discussed in [29]. Kan *et al.* [14] improve on Peled's ideas and manage to preserve all of LTL. To achieve this, they analyse the Büchi automaton that corresponds to the LTL formula to identify which part is stutter insensitive. With this information, they can reduce the state space in the appropriate places and preserve the validity of the LTL formula under consideration.

The recent work by Bønneland *et al.* [3] is close to ours in spirit, applying stubborn-set based POR to *reachability games*. Such games can be used for synthesis and for model checking reachability properties. Although the conditions on reduction they propose seem unaffected by the aforementioned issue with **D1**, unfortunately, their POR theory is nevertheless unsound, as we next illustrate.

In reachability games, player 1 tries to reach one of the *goal* states, while player 2 tries to avoid them. Bønneland *et al.* propose a condition **R** that guarantees that all goal states in the full game are also reachable in the reduced game. However, the reverse is not guaranteed: paths that do not contain a goal state are not necessarily preserved, essentially endowing player 1 with more power. Consider the (solitaire) reachability game depicted on the right, in which all edges belong to player 2 and the only goal state is indicated with grey. Player 2 wins the non-reduced game by avoiding the goal state via the edges labelled with $a$ and then $b$. However, $\{b\}$ is a stubborn set—according to the conditions of [3]—in the initial state, and the dashed transitions are thus eliminated in the reduced game. Hence, player 2 is forced to move the token to the goal state and player 1 wins in the reduced game. In the mean time, the authors of [3] confirmed and resolved the issue in [4].

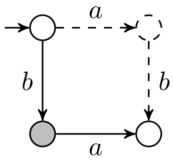

*Outline.* We give a cursory overview of parity games in Section 2. In Section 3 we introduce partial-order reduction for parity games, and we introduce a further improvement in Section 3.3. Section 4 briefly introduces the PBES fixed point logic, and in Section 5, we describe how to effectively implement parity-game based POR for PBESs. We present the results of our experiments of using parity-game based POR for PBESs in Section 6. We conclude in Section 7.

## 2 Preliminaries

Parity games are infinite-duration, two-player games played on a directed graph. The objective of the players, called *even* (denoted by $\Diamond$) and *odd* (denoted by $\Box$), is to win nodes in the graph.

**Definition 1.** *A* parity game *is a directed graph* $G = (V, E, \Omega, \mathcal{P})$*, where*
- $V$ *is a finite set of nodes, called the* state space*;*
- $E \subseteq V \times V$ *is a total edge relation;*
- $\Omega : V \to \mathbb{N}$ *is a function that assigns a* priority *to each node; and*
- $\mathcal{P} : V \to \{\Diamond, \Box\}$ *is a function that assigns a* player *to each node.*

We write $s \to t$ whenever $(s, t) \in E$. The set of successors of a node $s$ is denoted with $succ(s) = \{t \mid s \to t\}$. We use $\bigcirc$ to denote an arbitrary player and $\bar{\bigcirc}$ to denote its opponent.

A parity game is played as follows: initially, a token is placed on some node of the graph. The owner of the node can decide where to move the token; the token may be moved along one of the outgoing edges. This process continues ad infinitum, yielding an infinite path of nodes that the token moves through. Such a path is called a *play*. A play $\pi$ is won by player $\Diamond$ if the minimal priority that occurs infinitely often along $\pi$ is even. Otherwise, it is won by player $\Box$.

To reason about moves that a player may want to take, we use the concept of *strategies*. A strategy $\sigma_\bigcirc : V^+ \to V$ for player $\bigcirc$ is a partial function that determines where $\bigcirc$ moves the token next, after the token has passed through a finite sequence of nodes. More formally, for all sequences $s_1 \dots s_n$ such that $\mathcal{P}(s_n) = \bigcirc$, it holds that $\sigma_\bigcirc(s_1 \dots s_n) \in succ(s_n)$. If $s_n$ belongs to $\bar{\bigcirc}$, $\sigma_\bigcirc(s_1 \dots s_n)$ is undefined. A play $s_1, s_2, \dots$ is *consistent* with a strategy $\sigma$ if and only if $\sigma(s_1 \dots s_i) = s_{i+1}$ for all $i$ such that $\sigma(s_1 \dots s_i)$ is defined. A player $\bigcirc$ wins in a node $s$ if and only if there is a strategy $\sigma_\bigcirc$ such that all plays that start in $s$ and that are consistent with $\sigma_\bigcirc$ are won by player $\bigcirc$.

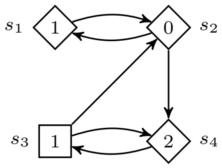

*Example 1.* Consider the parity game on the right. Here, priorities are inscribed in the nodes and the nodes are shaped according to their owner ($\Diamond$ or $\Box$). Let $\pi$ be an arbitrary, possibly empty, sequence of nodes. In this game, the strategy $\sigma_\Diamond$, partially defined as $\sigma_\Diamond(\pi s_1) = s_2$ and $\sigma_\Diamond(\pi s_2) = s_1$, is winning for $\Diamond$ in $s_1$ and $s_2$. After all, the minimal priority that occurs infinitely often along $(s_1 s_2)^\omega$ is 0, which is even. Player $\Box$ can win node $s_3$ with the strategy $\sigma_\Box(\pi s_3) = s_4$. Note that player $\Diamond$ is always forced to move the token from node $s_4$ to $s_3$. □

## 3 Partial-Order Reduction

In model checking, arbitrary interleaving of concurrent processes can lead to a combinatorial explosion of the state space. By extension, parity games that encode model checking problems for concurrent processes suffer from the same phenomenon. *Partial-order reduction* (POR) techniques help combat the blowup. Several variants of POR exist, such as *ample sets* [26], *persistent sets* [8] and *stubborn sets* [30,31]. The current work is based on Valmari's stubborn set theory as it can easily deal with nondeterminism [32].

### 3.1 Weak Stubborn Sets

Partial-order reduction relies on edge labels, here referred to as *events* and typically denoted with the letter $j$, to categorise the set of edges in a graph and determine independence of edges. In a typical application of POR, such events and edge labellings are deduced from a high-level syntactic description of the graph structure (see also Section 4). A *reduction function* subsequently uses these events when producing an equivalent *reduced* graph structure from the same high-level description. For now, we tacitly assume the existence of a set of events and edge labellings for parity games and refer to the resulting structures as *labelled parity games.*

**Definition 2.** *A* labelled parity game *is a triple* $L = (G, \mathcal{S}, \ell)$*, where* $G = (V, E, \Omega, \mathcal{P})$ *is a parity game,* $\mathcal{S}$ *is a non-empty set of events and* $\ell : \mathcal{S} \to 2^E$ *is an edge labelling.*

For the remainder of this section, we fix an arbitrary labelled parity game $L = (G, \mathcal{S}, \ell)$. We write $s \xrightarrow{j} t$ whenever $s \to t$ and $(s, t) \in \ell(j)$. The same notation extends to longer executions $s \xrightarrow{j_1 \cdots j_n} t$. We say an event $j$ is enabled in a node $s$, notation $s \xrightarrow{j}$, if and only if there is a transition $s \xrightarrow{j} t$ for some $t$. The set of all enabled events in a node $s$ is denoted with $enabled_G(s)$. An event $j$ is *invisible* if and only if $s \xrightarrow{j} t$ implies $\mathcal{P}(s) = \mathcal{P}(t)$ and $\Omega(s) = \Omega(t)$. Otherwise, $j$ is *visible.*

A *reduction function* indicates which edges are to be explored in each node, based on the events associated to the edges. Given some initial node $\hat{s}$, such a function induces a unique *reduced labelled parity game* as follows.

**Definition 3.** *Given a node* $\hat{s} \in V$ *and a* reduction function $r : V \to 2^{\mathcal{S}}$*. The* reduced labelled parity game *induced by* $r$ *and starting from* $\hat{s}$ *is defined as* $L_r = (G_r, \mathcal{S}, \ell_r)$*, where* $\ell_r(j) = \ell(j) \cap E_r$ *and* $G_r = (V_r, E_r, \Omega, \mathcal{P})$ *is such that:*
- $E_r = \{(s, t) \in E \mid \exists j \in r(s) : (s, t) \in \ell(j)\}$ *is the transition relation under* $r$*;*
- $V_r = \{s \mid \hat{s} E_r^* s\}$ *is the set of nodes reachable with* $E_r$*, where* $E_r^*$ *is the reflexive transitive closure of* $E_r$*.*

Note that a reduced labelled parity game is only well-defined when $r(s) \cap enabled_G(s) \neq \emptyset$ for every node $s \in V_r$; if this property does not hold, $E_r$ is not total. Even if totality of $E_r$ is guaranteed, the same node $s$ may be won by different players in $L$ and $L_r$ if no restrictions are imposed on $r$. The following conditions on $r$, as we will show, are sufficient to ensure both. Below, we say an event $j$ is a *key event* in $s$ iff for all executions $s \xrightarrow{j_1 \cdots j_n} s'$ such that $j_1 \notin r(s), \dots, j_n \notin r(s)$, we have $s' \xrightarrow{j}$. Key events are typically denoted $j_{\mathsf{key}}$.

**Definition 4.** *We say that a reduction function* $r : V \to 2^{\mathcal{S}}$ *is a* weak stubborn set *iff for all nodes* $s \in V$*, the following conditions hold*[1]*:*

[1] As noted before, the condition **D1** that we propose is stronger than the version in literature [30,33] since that one suffers from the *inconsistent labelling problem* [21] which also manifests itself in the parity game setting, see our technical report [24].

**D1** *For all* $j \in r(s)$ *and* $j_1 \notin r(s), \dots, j_n \notin r(s)$*, if* $s \xrightarrow{j_1} s_1 \xrightarrow{j_2} \cdots \xrightarrow{j_n} s_n \xrightarrow{j} s'_n$*, then there are nodes* $s', s_1, \dots, s'_{n-1}$ *such that* $s \xrightarrow{j} s' \xrightarrow{j_1} s'_1 \xrightarrow{j_2} \cdots \xrightarrow{j_n} s'_n$*. Furthermore, if* $j$ *is invisible, then* $s_i \xrightarrow{j} s'_i$ *for every* $1 \leq i < n$*.*
**D2w** $r(s)$ *contains a key event in* $s$*.*
**V** *If* $r(s)$ *contains an enabled visible event, then it contains all visible events.*
**I** *If an invisible event is enabled, then* $r(s)$ *contains an invisible key event.*
**L** *For every visible event* $j$*, every cycle in the reduced game contains a node* $s'$ *such that* $j \in r(s')$*.*

Below, we also use (weak) stubborn set to refer to the set of events $r(s)$ in some node $s$. First, note that every key event, which we typically denote by $j_{\mathsf{key}}$, in a node $s$ is enabled in $s$, by taking $n = 0$ in **D2w**; this guarantees totality of $E_r$. Condition **D1** ensures that whenever an enabled event is selected for the stubborn set, it does not disable executions not in $r(s)$. A stubborn set can never be empty, due to **D2w**. In a traditional setting where POR is applied on a transition system, the combination of **D1** and **D2w** is sufficient to preserve deadlocks. Condition **V** enforces that either all visible events are selected for the stubborn set, or none are. Condition **L** prevents the so called *action-ignoring problem*, where a certain event is never selected for the stubborn set and ignored indefinitely. Combined, **I** and **L** preserve plays with invisible events only.

We use the example below to further illustrate the purpose of—and need for—conditions **V**, **I** and **L**. In particular, the example illustrates that the winning player in the original game and the reduced game might be different if one of these conditions is not satisfied.

*Example 2.* See the three parity games of Figure 1. From left to right, these games show a reduced game under a reduction function satisfying **D1** and **D2w** but not **V**, **I** or **L**, respectively. In each case, we start exploration from the node called $\hat{s}$, using the reduction function to follow the solid edges; consequently, the winning strategy $\sigma_\Diamond$ for player $\Diamond$ in the original game is lost. □

Note that the games in Figure 1 are from a subclass of parity games called *weak solitaire*, illustrating the need for the identified conditions even in restricted

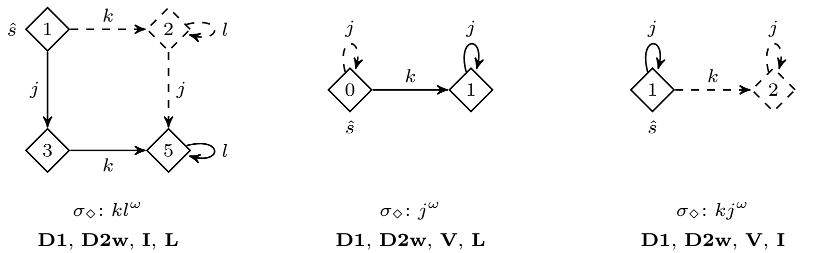

**Fig. 1.** Three games that show the winner is not necessarily preserved if we drop one of the conditions **V**, **I** or **L**, respectively. The dashed nodes and edges are present in the original game, but not in the reduced game. The edges taken from $\hat{s}$ by the winning strategy for player $\Diamond$ in the original game are indicated below each game.

settings. A game is *weak* if the priorities along all its paths are non-decreasing, *i.e.*, if $s \to t$ then $\Omega(s) \leq \Omega(t)$. A game is *solitaire* if only one player can make non-trivial choices. Weak solitaire games can encode the model checking of safety properties; solitaire games can capture logics such as LTL and ACTL$^*$.

Before we argue for the correctness of our POR approach in the next section, we finish with a small example that illustrates how our approach improves over existing methods for branching time logics.

*Example 3.* The conditions **C1-C3** of Gerth *et al.* [7] preserve LTL$_{-X}$ and are similar in spirit to our conditions. However, to preserve the branching structure, needed for preservation of CTL$_{-X}$, the following *singleton proviso* is introduced:
**C4** Either $enabled_G(s) \subseteq r(s)$ or $|r(s)| = 1$.
This extra condition can severely impact the amount of reduction achieved: consider the following two processes, where $n \geq 1$ is some large natural number.

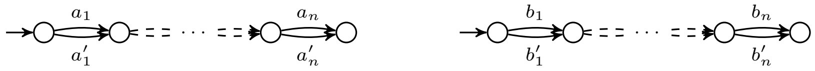

The cross product of these processes contains $(n+1)^2$ states. In the initial state, neither $\{a_1, a'_1\}$ nor $\{b_1, b'_1\}$ is a valid stubborn set, due to **C4**. However, the labelled parity game constructed using these processes and the $\mu$-calculus formula $\nu X.([-]X \wedge \mu Y.(\langle - \rangle Y \vee \langle a_n \rangle true))$, has a very similar shape that *can* be reduced by prioritising transitions that correspond to $b_i$ or $b'_i$ for some $1 \leq i \leq n$. Note that this formula cannot be represented in LTL; condition **C4** is therefore essential for the correctness. While several optimisations for CTL$_{-X}$ model checking under POR are proposed in [19], unlike our approach, those optimisations only work for certain classes of CTL$_{-X}$ formulas and not in general. □

### 3.2 Correctness

Condition **D2w** suffices, as we already argued, to preserve totality of the transition relation of the reduced labelled parity game. Hence, we are left to argue that the reduced game preserves and reflects the winner of the nodes of the original game; this is formally claimed in Theorem 1. We do so by constructing a strategy in the reduced game that mimics the winning strategy in the original game. The plays that are consistent with these two strategies are then shown to be *stutter equivalent*, which suffices to preserve the winner.

Fix a labelled parity game $L = (G, \mathcal{S}, \ell)$, a node $\hat{s}$, a weak stubborn set $r$ and the reduced labelled parity game $L_r = (G_r, \mathcal{S}, \ell_r)$ induced by $r$ and $\hat{s}$. We assume $r$ and $\hat{s}$ are such that $G_r$ has a finite state space. Below, $\omega$ is the set containing all natural numbers and the smallest infinite ordinal number.

**Definition 5.** *Let $\pi = s_0 s_1 s_2 \dots$ and $\pi' = t_0 t_1 t_2 \dots$ be two paths in $G$. We say $\pi$ and $\pi'$ are stutter equivalent, notation $\pi \triangleq \pi'$, if and only if one of the following conditions holds:*

- *$\pi$ and $\pi'$ are both finite and there exists a non-decreasing partial function $f : \omega \to \omega$, with $f(0) = 0$ and $f(|\pi|-1) = |\pi'|-1$, such that for all $0 \leq i < |\pi|$ and $i' \in [f(i), f(i+1))$, it holds that $\mathcal{P}(s_i) = \mathcal{P}(t_{i'})$ and $\Omega(s_i) = \Omega(t_{i'})$.*

– *$\pi$ and $\pi'$ are both infinite and there exists an unbounded, non-decreasing total function $f : \omega \to \omega$, with $f(0) = 0$, such that for all $i$ and $i' \in [f(i), f(i+1))$, it holds that $\mathcal{P}(s_i) = \mathcal{P}(t_{i'})$ and $\Omega(s_i) = \Omega(t_{i'})$.*

**Lemma 1.** *All infinite stutter equivalent paths have the same winner.*

In the lemmata below, we write $\to_r$ to stress which transition must occur in $G_r$.

**Lemma 2.** *Suppose $s_0 \xrightarrow{j_1} \cdots \xrightarrow{j_n} s_n \xrightarrow{j} s'_n$ for $j_1 \notin r(s_0), \ldots, j_n \notin r(s_0)$ and $j \in r(s_0)$. Then for some $s'_0, \ldots, s'_n$, both $s_0 \xrightarrow{j}_r s'_0 \xrightarrow{j_1} \cdots \xrightarrow{j_n} s'_n$ and $s_0 \ldots s_n s'_n \triangleq s_0 s'_0 \ldots s'_n$.*

**Lemma 3.** *Suppose $s_0 \xrightarrow{j_1} s_1 \xrightarrow{j_2} \ldots$ such that $j_i \notin r(s_0)$ for every $j_i$ occurring on this execution. Then, the following holds:*

– *If the execution ends in $s_n$, there exists a key event $j_{\mathsf{key}}$, and nodes $s'_0, \ldots, s'_n$ such that $s_n \xrightarrow{j_{\mathsf{key}}} s'_n$ and $s_0 \xrightarrow{j_{\mathsf{key}}}_r s'_0 \xrightarrow{j_1} \cdots \xrightarrow{j_n} s'_n$, and $s_0 \ldots s_n \triangleq s_0 s'_0 \ldots s'_n$.*
– *If the execution is infinite, there exists another execution $s_0 \xrightarrow{j_{\mathsf{key}}}_r s'_0 \xrightarrow{j_1} s'_1 \xrightarrow{j_2} \ldots$ for some key event $j_{\mathsf{key}}$ and $s_0 s_1 \cdots \triangleq s_0 s'_0 s'_1 \ldots$.*

We remark that Lemma 3 also holds for reduced labelled parity games that have an infinite state space, but where all the events are finitely branching. The proof of correctness, *viz.*, Theorem 1, uses the alternative executions described by Lemma 2 and 3. For full details, we refer to [24]; we here limit ourselves to sketching the intuition behind the application of these lemmata.

*Example 4.* The structure of Figure 2, in which parallel edges have the same label, visualises part of a game in which the solid edges labelled $j_1 j_2 j_3$ are part of a winning play for player □. This play is mimicked by path that follows the edges $j_{\mathsf{key}} j_2 j_1 j'_{\mathsf{key}} j_3$, drawn with dashes. The new play reorders the events $j_1$, $j_2$ and $j_3$ according to the construction of Lemma 2 and introduces the key events $j_{\mathsf{key}}$ and $j'_{\mathsf{key}}$ according to the construction of Lemma 3. □

The following theorem shows that partial-order reduction preserves the winning player in all nodes of the reduced game. Its proof is inspired by [30] and [2, Lemma 8.21], and uses the aforementioned lemmata.

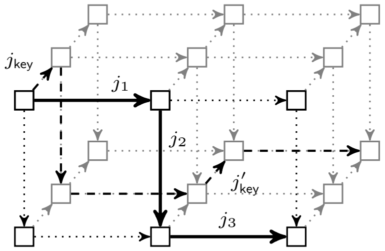

**Fig. 2.** Example of how $j_1$, $j_2$, $j_3$ is mimicked by introducing $j_{\mathsf{key}}$ and $j'_{\mathsf{key}}$ and moving $j_2$ to the front (dashed trace). Transitions that are drawn in parallel have the same label.

**Theorem 1.** *If $G_r$ has a finite state space then it holds that for every node $s$ in $G_r$, the winner of $s$ in $G_r$ is equal to the winner of $s$ in $G$.*

### 3.3 Optimising D2w

The theory we have introduced identifies and exploits rectangular structures in the parity game. This is especially apparent in condition **D1**. However, parity games obtained from model checking problems also often contain triangular structures, due to the (sometimes implicit) nesting of conjunctions and disjunctions, as the following example demonstrates.

*Example 5.* Consider the process $(a \parallel b) \cdot c$, in which actions $a$ and $b$ are executed in (interleaved) parallel, and action $c$ is executed upon termination of both $a$ and $b$. The $\mu$-calculus property $\mu X.([a]X \wedge [b]X \wedge \langle - \rangle \mathit{true})$, also expressible in LTL, expresses that the action $c$ must unavoidably be done within a finite number of steps; clearly this property holds true of the process. Below, the LTS is depicted on the left and a possible parity game encoding of our liveness property on this state space is depicted on the right. The edges in the labelled parity game that originate from the subformula $\langle - \rangle \mathit{true}$ are labelled with $d$.

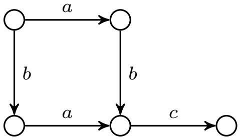

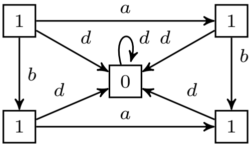

Whereas the state space of the process can be reduced by prioritising $a$ or $b$, the labelled parity game cannot be reduced due to the presence of a $d$-labelled edge in every node. For example, if $s$ is the top-left node in the labelled parity game, then $r(s) = \{a, d\}$ violates condition **D1**, since the execution $s \xrightarrow{bd}$ exists, but $s \xrightarrow{db}$ does not. □

In order to deal with games that contain triangular structures, we propose a condition that is weaker than **D2w**.

**D2t** There is an event $j \in r(s)$ such that for all $j_1 \notin r(s), \dots, j_n \notin r(s)$, if $s \xrightarrow{j_1} s_1 \xrightarrow{j_2} \cdots \xrightarrow{j_n} s_n$, then either $s_n \xrightarrow{j}$ or there are nodes $s', s'_1, \dots, s'_n$ such that $s \xrightarrow{j} s' \xrightarrow{j_1} s'_1 \xrightarrow{j_2} \cdots \xrightarrow{j_n} s'_n$ and for all $i$, $s_i = s'_i$ or $s_i \xrightarrow{j} s'_i$.

Theorem 1 holds even for reduction functions satisfying the weak stubborn set conditions in which condition **D2t** is used instead of condition **D2w**. The proof thereof resorts to a modified construction of a mimicking winning strategy that is based on Lemma 4, described below, instead of Lemma 3.

**Lemma 4.** *Let $r$ be a reduction function satisfying conditions **D1, D2t, V, I** and **L**. Suppose $s_0 \xrightarrow{j_1} s_1 \xrightarrow{j_2} \dots$ such that $j_i \notin r(s_0)$ for every $j_i$ occurring on this execution. Then, the following holds:*

- *If the execution ends in $s_n$, there exist a key event $j_{\mathsf{key}}$ and nodes $s'_0, \dots, s'_n$ such that:*
  - $s_n \xrightarrow{j_{\mathsf{key}}} s'_n$ *or* $s_n = s'_n$*; and*

- $s_0 \xrightarrow{j_{\mathsf{key}}}_r s_0' \xrightarrow{j_1} \cdots \xrightarrow{j_n} s_n'$ *and* $s_0 \dots s_n \triangleq s_0 s_0' \dots s_n'$.
- *If the execution is infinite, there exists another execution* $s_0 \xrightarrow{j_{\mathsf{key}}}_r s_0' \xrightarrow{j_1} s_1' \xrightarrow{j_2} \dots$ *and* $s_0 s_1 \dots \triangleq s_0 s_0' s_1' \dots$.

We remark that the concepts of triangular and rectangular structures bear similarities to the concept of weak confluence from [9].

## 4 Parameterised Boolean Equation Systems

Parity games are used, among others, to solve *parameterised Boolean equation systems* (PBESs) [10], which, in turn, are used to answer, *e.g.*, first-order modal $\mu$-calculus model checking problems [5]. In the remainder of this paper, we show how to apply POR in the context of solving a PBES (and, hence, the encoded decision problem). We first introduce PBESs and show how they induce labelled parity games.

Parameterised Boolean equation systems are sequences of fixed point equations over predicate formulae, *i.e.*, first-order logic formulae with second order variables. A PBES is given in the context of an abstract data type, which is used to reason about data. Non-empty data sorts of the abstract data type are typically denoted with the letters $D$ and $E$. The corresponding semantic domains are $\mathbb{D}$ and $\mathbb{E}$. We assume that sorts $B$ and $N$ represent the Booleans and the natural numbers respectively, and have $\mathbb{B}$ and $\mathbb{N}$ as semantic counterpart. The set of data variables is $\mathcal{V}$, and its elements are usually denoted with $d$ and $e$. To interpret expressions with variables, we use a *data environment* $\delta$, which maps every variable in $\mathcal{V}$ to an element of the corresponding sort. The semantics of an expression $f$ in the context of such an environment is denoted $[\![f]\!]\delta$. For instance, $[\![x < 2 + y]\!]\delta$ holds true iff $\delta(x) < 2 + \delta(y)$. To update an environment, we use the notation $\delta[v/d]$, which is defined as $\delta[v/d](d) = v$ and $\delta[v/d](d') = \delta(d')$ for all variables $d \neq d'$.

For lack of space, we only consider PBESs in *standard recursive form* [22,23], a normal form in which each right-hand side of an equation is a *guarded* formula instead of an arbitrary (monotone) predicate formula. We remark that a PBES can be rewritten to SRF in linear time, while the number of equations grows linearly in the worst case [23, Proposition 2].

Let $\mathcal{X}$ be a countable set of predicate variables. In the exposition that follows we assume for the sake of simplicity (but without loss of generality) that all predicate variables $X \in \mathcal{X}$ are of type $D$. We permit ourselves the use of non-uniformly typed predicate variables in our example.

**Definition 6.** *A guarded formula* $\phi$ *is a disjunctive or conjunctive formula of the form:*

$$\bigvee_{j \in J} \exists e_j{:}E_j.\, f_j \wedge X_j(g_j) \;\; \textit{or} \;\; \bigwedge_{j \in J} \forall e_j{:}E_j.\, f_j \Rightarrow X_j(g_j)$$

*where* $J$ *is an index set, each* $f_j$ *is a Boolean expression, referred to as* guard, *every* $e_j$ *is a (bound) variable of sort* $E_j$, *each* $g_j$ *is an expression of type* $D$ *and*

*each $X_j$ is a predicate variable of type $D$. A guarded formula $\phi$ is said to be* total *if for each data environment $\delta$, there is a $j \in J$ and $v \in \mathbb{E}_j$ such that $[\![f_j]\!]\delta[v/e_j]$ holds true.*

The denotational semantics of a guarded formula is given in the context of a data environment $\delta$ for interpreting data expressions and a *predicate environment* $\eta : \mathcal{X} \to 2^{\mathbb{D}}$, yielding an interpretation of $X_j(g_j)$ as the truth value $[\![g_j]\!]\delta \in \eta(X_j)$. Given a predicate environment and a data environment, a guarded formula induces a monotone operator on the complete lattice $(2^{\mathbb{D}}, \subseteq)$. By Tarski's theorem, least ($\mu$) and greatest ($\nu$) fixed points of such operators are guaranteed to exist.

**Definition 7.** *A* parameterised Boolean equation *in SRF is an equation that has the shape $(\mu X(d{:}D) = \phi(d))$ or $(\nu X(d{:}D) = \phi(d))$, where $\phi(d)$ is a total guarded formula in which $d$ is the only free data variable. A* parameterised Boolean equation system *in SRF is a sequence of parameterised Boolean equations in SRF, in which no two equations have the same left-hand side variable.*

Henceforward, let $\mathcal{E} = (\sigma_1 X_1(d{:}D) = \varphi_1(d)) \dots (\sigma_n X_n(d{:}D) = \varphi_n(d))$ be a fixed, arbitrary PBES in SRF, where $\sigma_i \in \{\mu, \nu\}$. The set of *bound predicate variables* of $\mathcal{E}$, denoted $\mathsf{bnd}(\mathcal{E})$, is the set $\{X_1, \dots, X_n\}$. If the predicate variables occurring in the guarded formulae $\varphi_i(d)$ of $\mathcal{E}$ are taken from $\mathsf{bnd}(\mathcal{E})$, then $\mathcal{E}$ is said to be *closed*; we only consider closed PBESs. Every bound predicate variable is assigned a *rank*, where $\mathsf{rank}_{\mathcal{E}}(X_i)$ is the number of alternations in the sequence of fixpoint symbols $\nu\sigma_1\sigma_2 \dots \sigma_i$. Observe that $\mathsf{rank}_{\mathcal{E}}(X_i)$ is *even* iff $\sigma_i = \nu$. We use the function $\mathsf{op}_{\mathcal{E}} : \mathsf{bnd}(\mathcal{E}) \to \{\vee, \wedge\}$ to indicate for each predicate variable in $\mathcal{E}$ whether the associated equation is disjunctive or conjunctive. As a notational convenience, we write $J_i$ to refer to the index set of the guarded formula $\varphi_i(d)$, and we assume that the index sets are disjoint for different equations.

The standard denotational fixed point semantics of a closed PBES associates a subset of $\mathbb{D}$ to each bound predicate variable (*i.e.*, their meaning is independent of the predicate environment used to interpret guarded formulae). For details of the standard denotational fixed point semantics of a PBES we refer to [10]. We forego the denotational semantics and instead focus on the (provably equivalent, see *e.g.* [23,6]) game semantics of a PBES in SRF.

**Definition 8.** *The* solution *to $\mathcal{E}$ is a mapping $[\![\mathcal{E}]\!] : \mathsf{bnd}(\mathcal{E}) \to 2^{\mathbb{D}}$, defined as $[\![\mathcal{E}]\!](X_i) = \{v \in \mathbb{D} \mid (X_i, v)$ is won by $\Diamond$ in $G_{\mathcal{E}}\}$, where $X_i \in \mathsf{bnd}(\mathcal{E})$ and $G_{\mathcal{E}}$ is the parity game associated to $\mathcal{E}$. The game $G_{\mathcal{E}} = (V, E, \Omega, \mathcal{P})$ is defined as:*

- *$V = \mathsf{bnd}(\mathcal{E}) \times \mathbb{D}$ is the set of nodes;*
- *$E$ is the edge relation, satisfying $(X_i, v) \to (X_j, w)$ for given $X_i$, $j \in J_i$, $v$ and $w$ if and only if for some $\delta$, both $[\![f_j]\!]\delta[v/d]$ and $w = [\![g_j]\!]\delta[v/d]$ hold;*
- *$\Omega((X_i, v)) = \mathsf{rank}_{\mathcal{E}}(X_i)$; and*
- *$\mathcal{P}((X_i, v)) = \Diamond$ iff $\mathsf{op}_{\mathcal{E}}(X_i) = \vee$.*

Note that the parity game $G_{\mathcal{E}}$ may have an infinite state space when $\mathbb{D}$ is infinite. In practice, we are often interested in the part of the parity game that is reachable from some initial node $(X, v)$; this is often (but not always) finite. This is illustrated by the following example.

*Example 6.* Consider the following PBES in SRF:

$$(\nu X(b{:}B) = (b \wedge X(\mathit{false})) \vee \exists n{:}N.\, n \leq 2 \wedge Y(b, \mathit{if}(b, n, 0)))$$
$$(\mu Y(b{:}B, n{:}N) = \mathit{true} \Rightarrow Y(\mathit{false}, 0))$$

The six nodes in the parity game which are reachable from $(X, \mathit{true})$ are depicted in Figure 3. The horizontally drawn edges all stem from the clause $\exists n{:}N.\, n \leq 2 \wedge Y(b, \mathit{if}(b, n, 0))$. Vertical edges stem from the clause $b \wedge X(\mathit{false})$ (on the left) or the clause $\mathit{true} \Rightarrow Y(\mathit{false}, 0)$ (on the right). The selfloop also stems from the clause $\mathit{true} \Rightarrow Y(\mathit{false}, 0)$. Player □ wins all nodes in this game, and thus $\mathit{true} \notin [\![\mathcal{E}]\!](X)$. □

As suggested by the above example, each edge is associated to (at least) one clause in $\mathcal{E}$. Consequently, we can use the index sets $J_i$ to event-label the edges emanating from nodes associated with the equation for $X_i$. We denote the set of all events in $\mathcal{E}$ by $\mathsf{evt}(\mathcal{E})$, defined as $\mathsf{evt}(\mathcal{E}) = \bigcup_{X_i \in \mathsf{bnd}(\mathcal{E})} J_i$. Event $j \in J_i$ is *invisible* if $\mathsf{rank}_{\mathcal{E}}(X_i) = \mathsf{rank}_{\mathcal{E}}(X_j)$ and $\mathsf{op}_{\mathcal{E}}(X_i) = \mathsf{op}_{\mathcal{E}}(X_j)$, and *visible* otherwise.

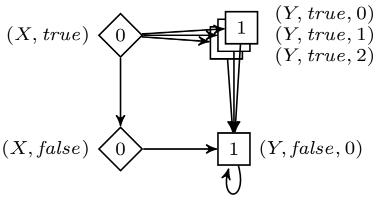

**Fig. 3.** Reachable part of the parity game underlying the PBES of Example 6, when starting from node $(X, \mathit{true})$.

**Definition 9.** *Let $G_{\mathcal{E}}$ be the parity game associated to $\mathcal{E}$. The labelled parity game associated to $\mathcal{E}$ is the structure $(G_{\mathcal{E}}, \mathsf{evt}(\mathcal{E}), \ell)$, where $G_{\mathcal{E}}$ is as defined in Def. 8, and, for $j \in J_i$, $\ell(j)$ is defined as the set $\{\langle (X_i, v), (X_j, w)\rangle \in E \mid [\![f_j]\!]\delta[v/d]$ holds true and $w = [\![g_j]\!]\delta[v/d]$ for some $\delta\}$.*

## 5 PBES Solving Using POR

A consequence of the partial-order reduction theorem is that a reduced parity game suffices for computing the truth value to $X(e)$ for a given PBES $\mathcal{E}$ with $X \in \mathsf{bnd}(\mathcal{E})$. However, **D1**, **D2w**/**D2t** and **L** are conditions on the (reduced) state space as a whole and, hence, hard to check locally. We therefore approximate these conditions in such a way that we can construct a stubborn set *on-the-fly.*

From hereon, let $\mathcal{E}$ be a PBES in SRF and $(G, \mathcal{S}, \ell)$, with $G = (V, E, \Omega, \mathcal{P})$, its labelled parity game. The most common local condition for **L** is the *stack proviso* $\mathbf{L}^S$ [26]. This proviso assumes that the state space is explored with *depth-first search* (DFS), and it uses the *Stack* that stores unexplored nodes to determine whether a cycle is being closed. If so, the node will be *fully expanded, i.e.*, $r(s) = \mathcal{S}$.

$\mathbf{L}^S$ For all nodes $s \in V_r$, either $\mathit{succ}_{G_r}(s) \cap \mathit{Stack} = \emptyset$ or $r(s) = \mathcal{S}$.

Locally approximating conditions **D1** and **D2w** requires a static analysis of the PBES. For this, we draw upon ideas from [17] and extend these to properly deal with non-determinism. To reason about which events are independent, we rely on the idea of *accordance.*

**Definition 10.** *Let $j, j' \in \mathcal{S}$. We define the* accordance *relations DNL, DNS, DNT and DNA on $\mathcal{S}$ as follows:*

- $j$ left-accords *with $j'$ if for all nodes $s, s' \in V$, if $s \xrightarrow{j'j} s'$, then also $s \xrightarrow{jj'} s'$. If $j$ does not left-accord with $j'$, we write $(j, j') \in DNL$.*
- $j$ square-accords *with $j'$ if for all nodes $s, s_1, s_2 \in V$, if $s \xrightarrow{j} s_1$ and $s \xrightarrow{j'} s_2$, then for some $s' \in V$, $s_1 \xrightarrow{j'} s'$ and $s_2 \xrightarrow{j} s'$. If $j$ does not square-accord with $j'$ we write $(j, j') \in DNS$.*
- $j$ triangle-accords *with $j'$ if for all nodes $s, s_1, s_2 \in V$, if $s \xrightarrow{j'} s_1$ and $s \xrightarrow{j} s_2$, then $s_2 \xrightarrow{j'} s_1$. If $j$ does not triangle-accord with $j'$ we write $(j, j') \in DNT$.*
- $j$ accords *with $j'$ if $j$ square-accords or triangle-accords with $j'$. If $j$ does not accord with $j'$ we write $(j, j') \in DNA$.*

Note that *DNL* and *DNT* are not necessarily symmetric. An illustration of the left-according, square-according and triangle-according conditions is given below.

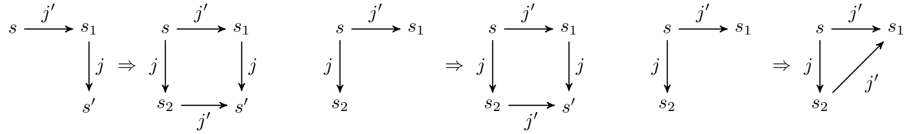

Accordance relations safely approximate the independence of events. The dependence of events, required for satisfying **D2w** can be approximated using Godefroid's *necessary enabling sets* [8].

**Definition 11.** *Let $j$ be an event that is disabled in some node $s$. A* necessary-enabling set *(NES) for $j$ in $s$ is any set $NES_s(j) \subseteq \mathcal{S}$ such that for every execution $s \xrightarrow{j_1 \cdots j_n j}$ there is at least one $j_i$ such that $j_i \in NES_s(j)$.*

For every node and event there might be more than one NES. In particular, every superset of a NES is also a NES. A larger-than-needed NES may, however, have a negative impact on the reduction that can be achieved. In a PBES with multiple parameters per predicate variable, computing a NES can be done by determining which parameters influence the validity of guards $f_j$ and which parameters are changed in the update functions $g_j$. A more accurate NES may be computed using techniques to extract a control flow from a PBES [15].

The following lemmata show how the accordance relations and necessary-enabling set can be used to implement conditions **D1**, **D2w** and **D2t**, respectively. A combination of Lemma 5 and 6 in a deterministic setting appeared as Lemma 1 in [17]. Note that as a notational convention we write $R(j)$ to denote the projection $\{j' \mid (j, j') \in R\}$ of a binary relation.

**Lemma 5.** *A reduction function $r$ satisfies **D1** in node $s \in V$ if for all $j \in r(s)$:*
- *if $j$ is disabled in $s$, then $NES_s(j) \subseteq r(s)$ for some $NES_s$; and*
- *if $j$ is enabled in $s$, then $DNL(j) \subseteq r(s)$.*

**Lemma 6.** *A reduction function $r$ satisfies **D2w** in a node $s \in V$ if there is an enabled event $j \in r(s)$ such that $DNS(j) \subseteq r(s)$.*

**Lemma 7.** *A reduction function $r$ satisfies **D2t** in a node $s$ if there is an enabled event $j \in r(s)$ such that $DNA(j) \subseteq r(s)$.*

More reduction can be achieved if a PBES is partly or completely 'deterministic', in which case some of the conditions can be relaxed. We say that an event $j$ is *deterministic*, denoted by $det(j)$, if for all nodes $t, t', t'' \in V$, if $t \xrightarrow{j} t'$ and $t \xrightarrow{j} t''$, then also $t' = t''$. This means event-determinism can be characterised as follows:

$$det(j) \text{ iff } [\![f_j]\!]\delta \text{ and } [\![f_j]\!]\delta' \text{ implies } [\![g_j]\!]\delta = [\![g_j]\!]\delta' \text{ for all } \delta, \delta' \text{ with } \delta(d) = \delta'(d).$$

The following lemma specialises Lemma 5 and shows how knowledge of deterministic events can be applied to potentially improve the reduction.

**Lemma 8.** *A reduction function $r$ satisfies **D1** in a node $s$ if for all $j \in r(s)$:*
- *if $j$ is disabled in $s$, then $NES_s(j) \subseteq r(s)$ for some $NES_s$; and*
- *if $det(j)$ and $j$ is enabled in $s$, then $DNS(j) \subseteq r(s)$ or $DNL(j) \subseteq r(s)$.*
- *if $\neg det(j)$ and $j$ is enabled in $s$, then $DNL(j) \subseteq r(s)$.*

Since relations *DNS* and *DNL* are incomparable we cannot decide *a priori* which should be used for deterministic events. However, Lemma 8 permits choosing one of the accordance sets on-the-fly. This choice can be made based on a heuristic function, similar to the function for NESs proposed in [17].

## 6 Experiments

We implemented the ideas from the previous section in a prototype tool, called `pbespor`, as part of the mCRL2 toolset [5]; it is written in C++. Our tool converts a given input PBES to a PBES in SRF, runs a static analysis to compute the accordance relations (see Section 5), and uses a depth-first exploration to compute the parity game underlying the PBES in SRF. The static analysis relies on an external SMT solver (we use Z3 in our experiments). To limit the amount of static analysis required and to improve the reduction, the implementation contains a rudimentary way of identifying whether the same event occurs in multiple PBES equations. Experiments are conducted on a machine with an Intel Xeon 6136 CPU @ 3 GHz, running mCRL2 with Git commit hash `dd36f98875`.

To measure the effectiveness of our implementation, we analysed the following mCRL2 models[2]: Anderson's mutual exclusion protocol [1], the dining philosophers problem, the gas station problem [11], Hesselink's handshake register [12], Le Lann's leader election protocol [18], Milner's Scheduler [20] and the Krebs cycle of ATP production in biological cells (model inspired by [25]). Most of these models are scalable. Each model is subjected to one or more requirements phrased as mCRL2's first-order modal $\mu$-calculus formulae. Where possible, Table 1 provides a CTL* formula that captures the essence of the requirement.

We analyse the effectiveness of our partial-order reduction technique by measuring the reduction of the size of the state space, and the time that is required to generate the state space. Since the static analysis that is conducted can require a non-neglible amount of time, we pay close attention to the various forms of static analysis that can be conducted. In particular, we compare the total time and effectiveness (in terms of reduction) of running the following static analysis:

[2] The models are archived online at `https://doi.org/10.5281/zenodo.3602969`.

**Table 1.** Runtime (analysis + exploration; in seconds) and number of states when exploring either the full state space or the reduced state space, for four different static analysis approaches. Figures printed in boldface indicate which of the additional static analyses is able to achieve the largest reduction over 'basic' (if any).

| | | full | | basic | | +DNL | | +NES | | +D2t | |
|---|---|---|---|---|---|---|---|---|---|---|---|
| model | property | nodes | time | nodes | time | nodes | time | nodes | time | nodes | time |
| gas station.c3 | $\exists\Diamond \mathit{accept}$ | 1 197 | 0.14 | 1 077 | 0.98 | 1 077 | 2.48 | 1 077 | 1.87 | **735** | 1.62 |
| gas station.c3 | $\exists\Box\exists\Diamond \mathit{pumping}$ | 1 261 | 0.15 | 967 | 0.98 | 967 | 2.61 | 967 | 1.99 | 967 | 1.72 |
| gas station.c3 | no deadlock | 1 197 | 0.18 | 735 | 0.95 | 735 | 2.52 | 735 | 2.04 | 735 | 1.52 |
| scheduler8 | no deadlock | 3 073 | 0.29 | 34 | 0.19 | 34 | 0.70 | 34 | 0.51 | 34 | 0.35 |
| scheduler10 | no deadlock | 15 361 | 1.65 | 42 | 0.25 | 42 | 0.90 | 42 | 0.65 | 42 | 0.42 |
| anderson.5 | $\forall\Diamond \mathit{cs}$ | 23 597 | 4.59 | 2 957 | 2.85 | 2 957 | 6.47 | 2 957 | 3.89 | 2 957 | 4.61 |
| hesselink | cache consistency | 91 009 | 5.28 | 82 602 | 8.19 | 83 602 | 12.12 | 81 988 | 9.00 | **71 911** | 8.51 |
| dining10 | no deadlock | 154 451 | 17.90 | 4 743 | 0.76 | 4 743 | 1.61 | 4 743 | 1.42 | 4 743 | 1.02 |
| krebs.3 | $\forall\Diamond \mathit{energy}$ | 238 877 | 24.38 | 232 273 | 24.59 | 232 273 | 25.62 | **209 345** | 21.73 | 232 273 | 24.42 |
| gas station.c6 | $\exists\Diamond \mathit{accept}$ | 186 381 | 38.00 | 150 741 | 40.55 | 150 741 | 45.50 | 150 741 | 43.16 | **75 411** | 21.40 |
| gas station.c6 | $\exists\Box\exists\Diamond \mathit{pumping}$ | 192 700 | 38.63 | 114 130 | 27.35 | 114 130 | 31.42 | 114 130 | 30.49 | 114 130 | 29.74 |
| gas station.c6 | no deadlock | 186 381 | 42.50 | 75 411 | 21.03 | 75 411 | 24.88 | 75 411 | 24.01 | 75 411 | 23.02 |
| scheduler14 | no deadlock | 344 065 | 53.14 | 58 | 0.37 | 58 | 1.31 | 58 | 0.97 | 58 | 0.61 |
| hesselink | $\forall\Box(\mathit{wr} \Rightarrow \exists\Diamond \mathit{fin})$ | 1 047 233 | 61.02 | 1 013 441 | 82.44 | 1 013 441 | 86.49 | 1 013 441 | 84.59 | **791 273** | 61.56 |
| hesselink | $\forall\Box(\mathit{wr} \Rightarrow \forall\Diamond \mathit{fin})$ | 1 047 232 | 70.14 | 791 320 | 64.05 | 791 374 | 66.53 | **749 936** | 62.98 | 791 268 | 67.59 |
| krebs.4 | $\forall\Diamond \mathit{energy}$ | 1 047 406 | 124.30 | 971 128 | 117.38 | 971 128 | 117.41 | **843 349** | 101.51 | 971 128 | 117.41 |
| lann.5 | consistent data | 818 104 | 142.38 | 818 104 | 170.18 | 818 104 | 175.87 | 818 104 | 177.78 | **761 239** | 155.22 |
| anderson.5 | no deadlock | 689 901 | 142.63 | 257 944 | 73.62 | 257 672 | 79.91 | 257 711 | 78.67 | 257 918 | 76.47 |
| lann.5 | no data loss | 1 286 452 | 199.74 | 453 130 | 73.28 | 453 130 | 77.31 | 453 130 | 74.40 | 453 130 | 75.52 |
| dining10 | $\forall\Box\forall\Diamond \mathit{eat}$ | 1 698 951 | 225.10 | 101 185 | 12.37 | 101 056 | 13.55 | 101 238 | 13.01 | 101 022 | 12.69 |
| anderson.7 | $\forall\Diamond \mathit{cs}$ | 3 964 599 | 1 331.91 | 124 707 | 63.83 | 124 707 | 73.87 | 124 707 | 68.67 | 124 707 | 69.68 |

– computing left-accordance (*DNL*) vs. over-approximating it with all events.
– computing a NES vs. over-approximating it with the set of all events $\mathcal{S}$.
– using **D2w** vs. the use of **D2t** (*i.e.*, use Lemma 6 vs. Lemma 7);

As a baseline for comparisons, we take a basic static analysis (over-approximated DNL, over-approximated NES, **D2w**), see column 'basic' in Table 1. In order to guarantee termination of the static analysis phase, we set a timeout of 200ms per formula that is sent to the solver. Table 1 reports on the statistics we obtained for exploring the full state space and the four possible POR configurations described above; the table is sorted with respect to the time needed for a full exploration. The time we list consists of the time needed to conduct the analysis plus the time needed for the exploration.

For most small instances, the time required for static analysis dominates any speed-up gained by the state space reduction. When the state spaces are larger, achieving a speed-up becomes more likely, while the highest overhead suffered by 'basic' is 55% (Hesselink, cache consistency). Significant reduction can be achieved even for non-trivial properties, such as 'lann.5' with 'no data loss'. Scheduler is an extreme case: its processes have very few dependencies, leading to an exponential reduction, both in terms of the state space size and in terms of time. In several cases, the use of a NES or **D2t** brings extra reduction (highlighted in bold). Moreover, the extra time required to conduct the additional analysis seems limited. The use of DNL, on the other hand, never pays off in our experiments; it even results in a slightly larger state space in two cases.

We note that there are also models, not listed in Table 1, where our static analysis does not yield any useful results and no reduction is achieved. Even if in such cases a reduction would be possible in theory, the current static analysis engines are unable to deal with the more complex data types often used in such models; *e.g.*, recursively defined lists or infinite sets, represented symbolically with higher-order constructions. This calls for further investigations into static analysis theories that can effectively deal with complex data.

Finally, we point out that in the case of, *e.g.*, the dining philosophers problem, the relative reduction under the 'no deadlock' property is much better than under the '$\forall\Box\forall\Diamond eat$' property. This demonstrates the impact properties can have on the reductions achievable, and it also points at a phenomenon we have not stressed in the current work, *viz.*, the impact of identifying events on the reductions achievable. We explain the phenomenon in the following example.

*Example 7.* Consider the LTS and the parity game on the right. The parity game encodes the property $\nu X.([-]X \wedge \forall i.\, \mu Y.([\overline{a_i}]Y \wedge \langle - \rangle \mathit{true}))$, which is equivalent to $\forall\Box\Diamond a_i$, on this LTS. The event $xy$ represents the transition from fixpoint $X$ into $Y$, which does not involve an action from the LTS. Note that the complete state space is encoded in the fixpoint $X$. Due to the absence of some transitions in the part of the state space encoded in fixpoint $Y$, neither $a_1$ nor $a_2$ is according with $xy$. Hence, the only stubborn set in the initial node is $\{a_1, a_2, xy\}$, which yields no reduction. $\Box$

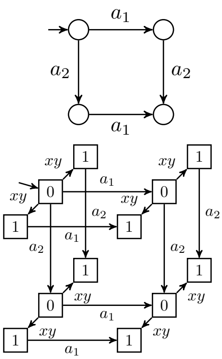

Improving the event identification procedure can yield more reduction. For instance, if, for each $i$ (bound in the universal quantifier), a different event $xy_i$ is created, then both $a_1, xy_2$ and $a_2, xy_1$ will be according. If we disregard the visibility of $xy_1$ and $xy_2$, four nodes can be eliminated.

## 7 Conclusion

We have presented an approach for applying partial-order reduction on parity games. This has two main advantages over POR applied on LTSs or Kripke structures: our approach supports the full modal $\mu$-calculus, not just a fragment thereof, and the potential for reduction is greater, because we do not require a singleton proviso. Furthermore, we have shown how the ideas can be implemented with PBESs as a high-level representation. In future work, we aim to gain more insight into the effect of identifying events across PBES equations in several ways. We also want to investigate the possibility of solving a reduced parity game while is it being constructed. In certain cases, one may be able to decide the winner of the original game from this partial solution.

## References

[1] Anderson TE (1990). The Performance of Spin Lock Alternatives for Shared-Memory Multiprocessors. IEEE Transactions on Parallel & Distributed Systems.

[2] Baier, C., Katoen, J.P.: Principles of model checking. MIT Press (2008)

[3] Bønneland, F.M., Jensen, P.G., Larsen, K.G., Muñiz, M.: Partial Order Reduction for Reachability Games. In: CONCUR 2019. vol. 140, pp. 23:1–23:15 (2019). 10.4230/LIPIcs.CONCUR.2019.23

[4] Bønneland, F.M., Jensen, P.G., Larsen, K.G., Mũniz, M., Srba, J.: Stubborn Set Reduction for Two-Player Reachability Games. arXiv:1912.09875 (2019)

[5] Bunte, O., Groote, J.F., Keiren, J.J.A., Laveaux, M., Neele, T., de Vink, E.P., Wesselink, J.W., Wijs, A.W., Willemse, T.A.C.: The mCRL2 Toolset for Analysing Concurrent Systems: Improvements in Expressivity and Usability. In: TACAS 2019. LNCS, vol. 11428, pp. 21–39 (2019). 10.1007/978-3-030-17465-1_2

[6] Cranen, S., Luttik, B., Willemse, T.A.C.: Proof graphs for parameterised Boolean equation systems. In: CONCUR 2013. LNCS, vol. 8052, pp. 470–484 (2013). 10.1007/978-3-642-40184-8_33

[7] Gerth R, Kuiper R, Peled D, Penczek W (1999). A Partial Order Approach to Branching Time Logic Model Checking. Information and Computation.

[8] Godefroid, P.: Partial-Order Methods for the Verification of Concurrent Systems, LNCS, vol. 1032. Springer (1996). 10.1007/3-540-60761-7

[9] Groote, J.F., Sellink, M.P.A.: Confluence for process verification. Theoretical Computer Science **170**(1-2), 47–81 (1996). 10.1016/s0304-3975(96)00175-2

[10] Groote JF, Willemse TAC (2005). Parameterised boolean equation systems. Theoretical Computer Science.

[11] Heimbold D, Luckham D (1985). Debugging ada tasking programs. IEEE Software.

[12] Hesselink WH (1998). Invariants for the construction of a handshake register. Inf. Process. Lett..

[13] Ip, C.N., Dill, D.L.: Better verification through symmetry. Formal Methods in System Design **9**(1-2), 41–75 (1996). 10.1007/BF00625968

[14] Kan S, Huang Z, Chen Z, Li W, Huang Y (2017). Partial order reduction for checking LTL formulae with the next-time operator. Journal of Logic and Computation.

[15] Keiren, J.J.A., Wesselink, J.W., Willemse, T.A.C.: Liveness Analysis for Parameterised Boolean Equation Systems. In: ATVA 2014. LNCS, vol. 8837, pp. 219–234 (2014). 10.1007/978-3-319-11936-6_16

[16] Kozen D (1982). Results on the propositional $$\mu $$-calculus. Theoretical Computer Science.

[17] Laarman, A., Pater, E., van de Pol, J., Hansen, H.: Guard-based partial-order reduction. STTT **18**(4), 427–448 (2016). 10.1007/s10009-014-0363-9

[18] Lann GL (1977). Distributed systems - towards a formal approach. IFIP.

[19] Liebke, T., Wolf, K.: Taking Some Burden Off an Explicit CTL Model Checker. In: Petri Nets 2019. LNCS, vol. 11522, pp. 321–341 (2019). 10.1007/978-3-030-21571-2_18

[20] Milner, R.: A Calculus of Communicating Systems, LNCS, vol. 92. Springer (1980)

[21] Neele, T., Valmari, A., Willemse, T.A.C.: The Inconsistent Labelling Problem of Stutter-Preserving Partial-Order Reduction. In: FoSSaCS 2020. LNCS, vol. 2077 (2020). 10.1007/978-3-030-45231-5_25

[22] Neele, T., Willemse, T.A.C., Groote, J.F.: Solving Parameterised Boolean Equation Systems with Infinite Data Through Quotienting. In: FACS 2018. LNCS, vol. 11222, pp. 216–236 (2018). 10.1007/978-3-030-02146-7_11

[23] Neele T, Willemse TAC, Groote JF (2020). Finding compact proofs for infinite-data parameterised Boolean equation systems. Science of Computer Programming.

[24] Neele, T., Willemse, T.A.C., Wesselink, W.: Partial-Order Reduction for Parity Games with an Application on Parameterised Boolean Equation Systems (Technical Report). Tech. rep., Eindhoven University of Technology (2019)

[25] Pelánek, R.: BEEM: Benchmarks for Explicit Model Checkers. In: SPIN 2007. LNCS, vol. 4595, pp. 263–267 (2007). 10.1007/978-3-540-73370-6_17

[26] Peled, D.: All from One, One for All: on Model Checking Using Representatives. In: CAV 1993. LNCS, vol. 697, pp. 409–423 (1993). 10.1007/3-540-56922-7_34

[27] Peled D (1996). Combining partial order reductions with on-the-fly model-checking. FMSD.

[28] Ramakrishna, Y.S., Smolka, S.A.: Partial-Order Reduction in the Weak Modal Mu-Calculus. In: CONCUR 1997. LNCS, vol. 1243, pp. 5–24 (1997). 10.1007/3-540-63141-0_2

[29] Siegel, S.F.: What’s Wrong with On-the-Fly Partial Order Reduction. In: CAV 2019. LNCS, vol. 11562, pp. 478–495 (2019). 10.1007/978-3-030-25543-5_27

[30] Valmari A (1992). A Stubborn Attack on State Explosion. Formal Methods in System Design.

[31] Valmari, A.: The state explosion problem. In: ACPN 1996. LNCS, vol. 1491, pp. 429–528 (1996). 10.1007/3-540-65306-6_21

[32] Valmari, A.: Stubborn Set Methods for Process Algebras. In: POMIV 1996. DIMACS, vol. 29, pp. 213–231 (1997). 10.1090/dimacs/029/12

[33] Valmari A, Hansen H (2017). Stubborn Set Intuition Explained. ToPNoC.

